# Two Cases of Acute Cholecystitis and Symptomatic Choledocholithiasis in Two Women Less than 40-Years-of-Age with Hormonal Intrauterine Devices

**DOI:** 10.1155/2018/2390213

**Published:** 2018-11-21

**Authors:** Helen M. Shields, Hasrat Sidhu

**Affiliations:** ^1^Helen Shields, M.D., Division of Gastroenterology, Hepatology and Endoscopy, Department of Medicine, Brigham and Women's Hospital and Harvard Medical School, USA; ^2^Hasrat Sidhu, M.B.B.S, Dayanand Medical College and Hospital, Ludhiana, Punjab, India

## Abstract

Levonorgestrel uterine implants are accepted as a safe and efficacious method of contraception. One of the two major health side effects in a large controlled study of subcutaneous hormonal implants with levonorgestrel was a significant increase in gallbladder disease. Gallbladder hypomotility is recognized as a side effect of the levonorgestrel (progesterone). We recently saw on a Gastroenterology Consult Service, two women under 40-years-of-age who had been transferred from outside hospitals with acute cholecystitis with symptomatic choledocholithiasis. Both required Endoscopic Retrograde Cholangiopancreatography and sphincterotomies in addition to laparoscopic cholecystectomies. Both had hormonal (levonorgestrel-releasing) intrauterine devices in place for contraception. Although one patient had a family history of gallstones, the other did not. Both were nonobese, young women patients. We were struck by the coincidence of seeing two such patients. Few articles in the medical literature detail the clinical risks of gallstone disease in patients with hormonal (levonorgestrel-releasing) intrauterine devices. Our experiences with these two patients led us to believe that patients with risk factors for gallstone disease, such as a positive family history, ethnic predisposition, or obesity, should be warned of possible problems, not only with gallbladder disease, but also of common duct stones.

## 1. Introduction

Gallstone disease is common worldwide affecting 10–15% of the adult population [1]. Studies have shown that gallstone formation is multifactorial. Some risk factors include ethnicity, age, gender, family history, diet, and obesity. Women are twice as likely to develop gallstones compared to men [1]. Estrogen and progesterone are largely responsible for the difference in the incidence. Estrogen causes an increase in cholesterol secretion into bile leading to its supersaturation whereas progesterone impairs gallbladder motility causing bile stasis. Pregnancy, oral contraceptives, and hormonal therapy lead to an increase in the serum levels of these hormones and are established risk factors for gallstones [2–4]. Women using levonorgestrel-releasing uterine contraceptive subcutaneous implant showed a significant increase in gallbladder disease as compared to women using nonhormonal intrauterine contraceptive devices or surgical sterilization using a controlled cohort methodology [5]. We describe two nonobese, young women patients with hormonal (levonorgestrel-releasing) intrauterine devices with both common duct stones and acute cholecystitis.

We present two cases of symptomatic choledocholithiasis and acute cholecystitis seen by us (H.M.S. and H.S.) on a busy Gastroenterology Inpatient Consult Service at a tertiary care hospital in the fall of 2017 in nonobese women under 40-years-of-age who were transferred from outside hospitals. Both patients had hormonal-releasing intrauterine devices in place for contraception. Our memorable experiences with these two young women patients led us to report these two cases given that few articles in the medical literature discuss the occurrence of both acute cholecystitis and choledocholithiasis in patients with hormonal intrauterine devices.

## 2. Case Presentations

### 2.1. Case # 1

**Figure 1:**
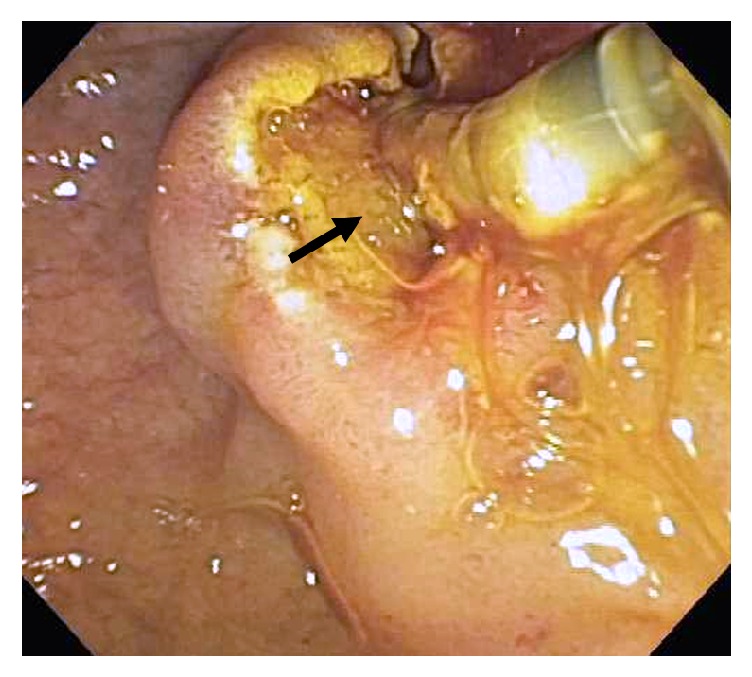
Case # 1, Sludge (arrow) coming from ampulla after sphincterotomy during ERCP.

Our first case was of a 39-year-old woman with two children with a history of ulcerative proctitis who was transferred to our hospital with a complaint of epigastric and right upper quadrant pain that began the evening before after eating a coconut macaroon and persisted with four episodes of vomiting bile. She was initially seen at another Boston Hospital where a CT of the Abdomen and Pelvis demonstrated a gallstone in the neck of the gallbladder, a white count of 11,900K/ul, and a Lactate of 2.2. mmol/L. She was given Morphine 4 mg IV, Zofran 4 mg IV and 1 liter of Normal Saline and Ceftriaxone 1 gram, and 500 mg PO of Flagyl prior to her transfer to our hospital. At our hospital, her laboratory studies revealed an elevated white count of 10,4600 with 88% Neutrophils, elevated Total Bilirubin of 1.5 mg/dl and AST of 66 U/L, and a normal Lipase of 34 U/L. The patient had cholelithiasis diagnosed several weeks before at our hospital, after an Ultrasound of the Abdomen was performed during an Emergency Room (ER) visit for episodic epigastric pain radiating to the right upper quadrant for one and a half weeks. The episodes of pain were triggered by rich or fatty foods. Following her ER visit, she was referred the same day to a General Surgeon who scheduled her for an elective laparoscopic cholecystectomy. Her family history was positive for history of gallstones in a brother. A Skyla hormonal intrauterine device (IUD) with 13.5 mg of Levonorgestrel, a synthetic form of the hormone progesterone, had been placed on 9/16/17, five weeks before the onset of symptoms. With a Skyla, the levonorgestrel release rate is 14 mcg/24 hours after 24 days and 5 mcg/24 hours after 3 years. An ERCP and Sphincterotomy were performed. The ERCP was noted to be difficult and complex. The common bile duct was mildly dilated without evidence of intrahepatic duct dilatation and no stones. Sludge was removed from the duct (Figure 1). Following the ERCP, the patient developed, later that evening, severe epigastric pain with an amylase of 3128 U/L and Lipase of 11030 U/L was diagnosed as Post-ERCP pancreatitis and she was hospitalized for three additional days.

A laparoscopic cholecystectomy was performed a month later. The gallbladder was noted to be large, distended with signs of acute inflammation and edema. Pathology report showed chronic cholecystitis. Patient did well and was discharged on the same day.

At her postoperative visit, good healing of laparoscopic incision was noted. The patient had the Skyla IUD removed three months later due to interest in becoming pregnant again.

### 2.2. Case # 2

A 37-year-old woman with two young children and a history of endometriosis was transferred from an outside hospital to our hospital for abnormal liver function tests and a CT of the Abdomen and Pelvis showing a common bile duct stone. The patient had had intermittent right upper quadrant discomfort for the past year and a half. She was evaluated for this previously at an outside hospital, but the pain was dismissed as musculoskeletal in origin. At our hospital, the patient complained of two days of persistent right upper quadrant pain with nausea and loss of appetite. The outside hospital laboratory tests showed elevated liver function tests including Total Bilirubin of 6.0 mg/dl, Alkaline phosphatase 171 U/L, ALT of 970 U/L, and AST of 706 U/L. On physical examination, the patient was noted to have scleral icterus. Her abdomen was soft, nondistended with tenderness to palpation in the right upper quadrant and a positive Murphy's sign. Laboratory tests from our hospital on 11/8/17 showed a Total Bilirubin of 6.9 mg/dl (ref. range <1.2 mg/dl), Direct Bilirubin of 6.2 mg/dl (ref. range 0-0.3 mg/dl), Alkaline Phosphatase 166 U/L (ref. range 37-116 U/L), ALT 599 U/L (ref. range 0-40 U/L), and AST 301 U/L (ref. range 9-32 U/L)

**Figure 2:**
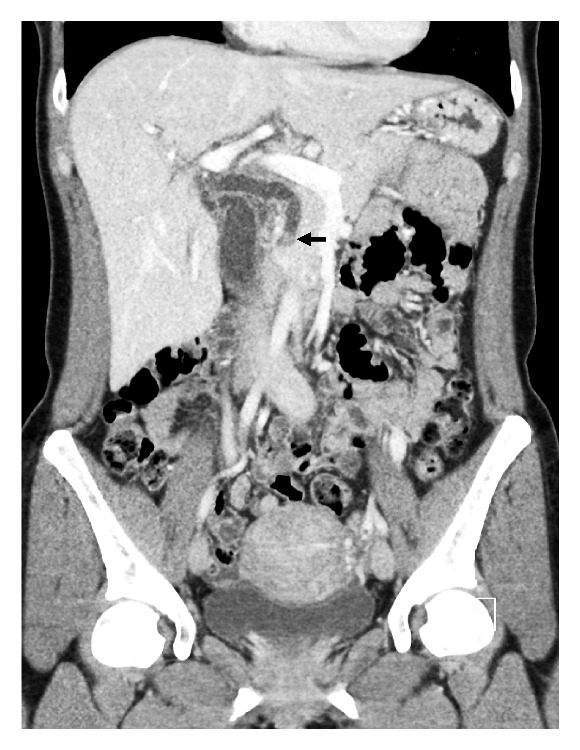
Case # 2, Dilated common bile duct with distal common bile duct stone (arrow), CT of Abdomen.

**Figure 3:**
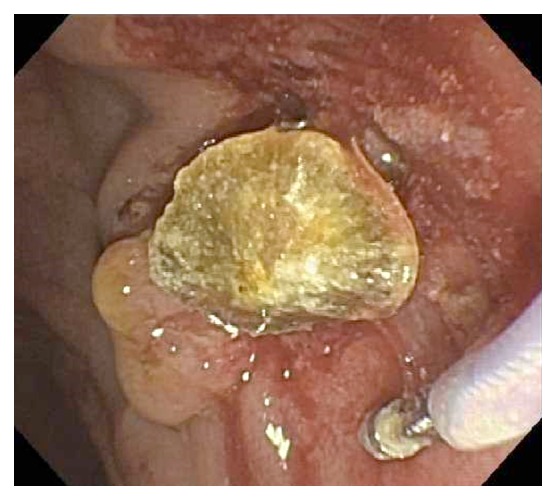
Case # 2, Gallstone in duodenum removed by ERCP sphincterotomy, balloon dilation, and basket procedure.

**Figure 4:**
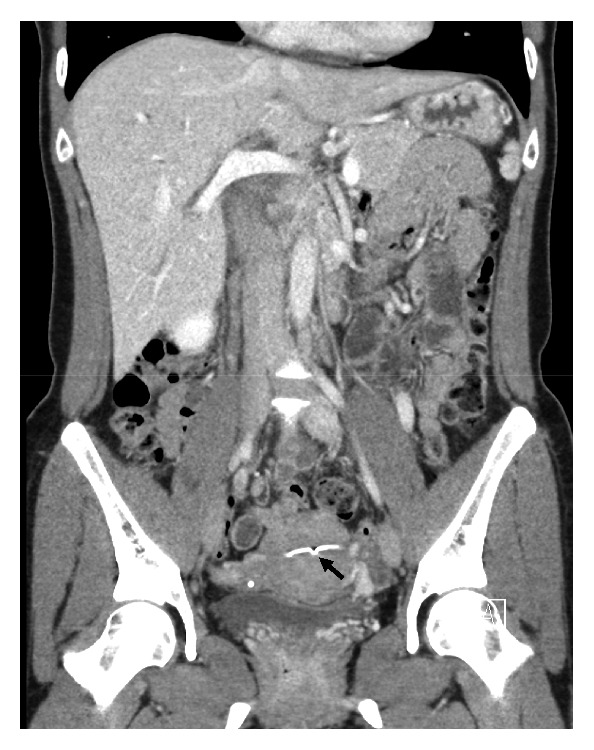
Case # 2, Mirena hormonal intrauterine device in uterus (arrow), CT of Abdomen.

Review of the outside contrast enhanced CT of abdomen and pelvis showed dilatation of the common bile duct measuring 9 mm with a filling defect in the distal common bile duct consistent with choledocholithiasis (Figure 2) and mild mucosal enhancement of the gallbladder with pericholecystic inflammation compatible with a diagnosis of acute cholecystitis. The patient was taken for an endoscopic retrograde cholangiopancreatography (ERCP). The cholangiogram revealed a moderately dilated common bile duct with a smooth tapering distally and a single large obstructive stone. A biliary sphincterotomy was performed followed by balloon sphincterotomy and complex balloon and basket guided removal were performed to fragment the stone into three large parts and remove it successfully (Figure 3). In the pelvis, a fibroid uterus with intrauterine device was noted. Mirena (levonorgestrel 20 mcg/24 hours for 5 years) intrauterine device had been placed two years earlier in 2015 (Figure 4).

The patient underwent a laparoscopic cholecystectomy without complications. Pathology of the gallbladder showed chronic cholecystitis. The patient did well in the postoperative period and developed no complications. She was discharged home two days after surgery. At her routine Surgery Clinic follow-up, a month later, the patient felt well with no pain. She was given permission to return to work.

## 3. Discussion

We were struck by seeing two young women with normal BMIs on the Gastroenterology Inpatient Consult Service at a tertiary care hospital, this past fall with acute cholecystitis and choledocholithiasis who both had hormonal intrauterine devices. Each was transferred from an outside hospital for a therapeutic ERCP when it was noted that each had had an imaging study showing choledocholithiasis. Both also had acute cholecystitis by imaging and physical examination. We believe that our concentrated and memorable experiences with these two young women patients deserve to be recognized by gastroenterologists who are taking care of patients with hormonal intrauterine devices for contraception.

Meirik, Farley and Sivin published a concurrent cohort study of 5 years' duration in 2001 which followed women in eight developing countries who were using Norplant subcutaneous implants, a set of six flexible closed capsules each containing 36 mg of the progestin, levonorgestrel. The capsules are inserted in a superficial plane beneath the skin of the upper arm. In the study, Norplant patients (n= 7977) were compared to women choosing nonhormonal intrauterine devices (n= 6625) or surgical sterilization (n=1419). Participants were interviewed semi-annually and followed up for five years. The overall follow-up rate was 94.6 % and 78,323 woman-years of observation were accumulated. One of the two significant excess risks of serious morbidity that were detected for Norplant (levonorgestrel) users compared to non-users was a significant increase in gallbladder disease. The incidence of gallbladder disease was significantly higher in women who were Norplant users than in the controls (rate ratio 1.52, 95 % confidence interval [CI] 1.02, 2.27). The other excessive risk was the presence of hypertension. The gallbladder diseases noted in this study were gallstones, acute cholecystitis, and chronic cholecystitis with a significant p value of p=0.002 compared to the control group. No mention of common duct stones or choledocholithiasis is made in this paper. Individual patient blogs have also noted gallstones in patients with hormonal intrauterine devices that contain the progestin, levonorgestrel.

The incidence of choledocholithiasis has been found to be 3%-10% in patients undergoing cholecystectomy [6]. The incidence of choledocholithiasis in patients with cholelithiasis increases with age. Women have a higher risk of developing gallstones due to the influence of estrogen and progesterone. Biliary sludge has frequently been reported during pregnancy and noted to disappear after delivery [7]. The proposed mechanism is an increase in cholesterol secretion leading to supersaturation of bile and formation of gallstones by action on estrogen receptors in the gallbladder and liver [8].

Gallbladder hypomotility leading to stasis of bile is another mechanism which predisposes to formation of stones. The presence of progesterone receptors in the gallbladder wall was associated with a decreased percentage of ejection compared with both healthy control subjects and patients whose gallbladders were receptor-negative [9]. Animal studies have suggested progesterone to be interfering with G-protein signaling pathway leading to impaired motility [10, 11]. One study found progesterone to inhibit L-type calcium channels in the gallbladder smooth muscle cells [12].

These physiologic studies help explain the increased risk of gallbladder disease seen in women using subcutaneous hormonal implants for contraception [5]. In our cases with levonorgestrel-releasing hormonal intrauterine devices, the two women started experiencing symptoms between a few weeks to two years after the insertion of a hormonal intrauterine device. Even though the systemic absorption from hormonal intrauterine devices is low, reaching plasma levels less than those compared to skin implants [5] and oral contraceptives, the long-term use of hormonal intrauterine devices could predispose to developing gallbladder disease particularly in women with additional risk factors such as family history which was present in one of our two patients. Both of our women patients had normal BMIs (Case # 1 BMI = 20.33 and Case # 2 BMI = 21.9), indicating that obesity did not play a role in their risk for gallstones.

In summary, we consulted on two nonobese, young women patients with hormonal (levonorgestrel-releasing) intrauterine devices on a busy Gastroenterology Inpatient Consult Service who had choledocholithiasis and acute cholecystitis. We wish to bring to the attention of gastroenterologists and therapeutic endoscopists the increased risk, not only for gallstones, acute and chronic cholecystitis, as previously reported with subcutaneous implants of the progestin, levonorgestrel [5], the same hormone in hormonal-releasing intrauterine implants, but also for common bile duct stones in two nonobese, young women patients.

We look forward to a prospective controlled study being performed on women with hormonal-releasing intrauterine devices versus nonhormonal intrauterine devices to confirm the increased risk, not only for gallstones and acute cholecystitis, but also for choledocholithiasis.
